# HIV Care Outcomes among Hispanics/Latinos with Diagnosed HIV in the United States by Place of Birth-2015–2018, Medical Monitoring Project

**DOI:** 10.3390/ijerph17010171

**Published:** 2019-12-25

**Authors:** Hanna B. Demeke, Qingwei Luo, Ruth E. Luna-Gierke, Mabel Padilla, Gladys Girona-Lozada, Sandra Miranda-De León, John Weiser, Linda Beer

**Affiliations:** 1 The National Center for HIV/AIDS, Viral Hepatitis, STD, and TB Prevention (NCHHSTP), Centers for Disease Control and Prevention (CDC), Atlanta, GA 30329, USA; 2 ICF Assigned to DHAP, NCHHSTP, CDC, Atlanta, GA 30329, USA; 3 Puerto Rico Department of Health, San Juan, PR 00936, USA

**Keywords:** human immunodeficiency virus, Hispanic/Latino, Puerto Rico, migrants and immigrants, HIV medical care, ART prescription, retention in medical care, viral suppression, Ryan White HIV/AIDS Program

## Abstract

Relocation from one’s birthplace may affect human immunodeficiency virus (HIV) outcomes, but national estimates of HIV outcomes among Hispanics/Latinos by place of birth are limited. We analyzed Medical Monitoring Project data collected in 2015–2018 from 2564 HIV-positive Hispanic/Latino adults and compared clinical outcomes between mainland US-born (referent group), Puerto Rican (PR-born), and those born outside the United States (non-US-born). We reported weighted percentages of characteristics and used logistic regression with predicted marginal means to examine differences between groups (*p* < 0.05). PR-born Hispanics/Latinos were more likely to be prescribed antiretroviral therapy (ART) (94%) and retained in care (94%) than mainland-US-born (79% and 77%, respectively) and non-US-born (91% and 87%, respectively) Hispanics/Latinos. PR-born Hispanics/Latinos were more likely to have sustained viral suppression (75%) than mainland-US-born Hispanics/Latinos (57%). Non-US-born Hispanics/Latinos were more likely to be prescribed ART (91% vs. 79%), retained in care (87% vs. 77%), and have sustained viral suppression (74% vs. 57%) than mainland-US-born Hispanics/Latinos. Greater Ryan White HIV/AIDS-funded facility usage among PR-born, better mental health among non-US-born, and less drug use among PR-born and non-US-born Hispanics/Latinos may have contributed to better HIV outcomes. Expanding programs with comprehensive HIV/AIDS services, including for mental health and substance use, may reduce HIV outcome disparities among Hispanics/Latinos.

## 1. Introduction

Human immunodeficiency virus (HIV) continues to be a serious threat to the health of Hispanic/Latino communities. Hispanics/Latinos represented 18% of the US population in 2015 but accounted for 27% of new HIV diagnoses in 2016 [1]. Despite overall improvements in HIV outcomes in the United States, disparities still exist between Hispanic and non-Hispanic persons with diagnosed HIV. A lower proportion of Hispanics/Latinos than non-Hispanic whites with diagnosed HIV received HIV medical care and had suppressed viral load [2]. Viral suppression is dependent on timely diagnosis and linkage to and retention in HIV medical care. Overall, Hispanics/Latinos with diagnosed HIV were more likely to enter HIV care late in the disease course, often with a concurrent acquired immunodeficiency syndrome (AIDS) diagnosis, and less likely to be linked to medical care compared with non-Hispanic whites [3,4]. Improving access to care, retention in care, and viral suppression among Hispanic/Latino populations with diagnosed HIV is needed to reduce disparities and achieve national prevention goals [5].

Hispanics/Latinos born outside of the mainland United States comprised 61% of Hispanics/Latinos with diagnosed HIV in the United States in 2003–2006 [6]. Of people who received a diagnosis of HIV in the United States in 2007–2010, 42% of Hispanics/Latinos were born outside of the United States and its territories, compared with only 3% of non-Hispanic whites and 10% of non-Hispanic blacks born outside of the United States and its territories [7]. Analyses that do not stratify estimates for Hispanics/Latinos by place of birth cannot identify similarities and differences in important health outcomes and associated factors within the Hispanic/Latino community.

Non-US-born persons with diagnosed HIV were more likely to be prescribed antiretroviral therapy (ART), retained in care, and virally suppressed, compared with US-born persons [8]. However, national estimates that describe HIV care outcomes among Hispanics/Latinos by place of birth are limited [4]. A preliminary analysis that categorized Hispanics/Latinos born in Puerto Rico (PR) with the US-born group demonstrated no significant differences in HIV care outcomes. As a result, we analyzed more recent data to describe social determinants of health, behavioral characteristics, and HIV care outcomes by place of birth among Hispanic/Latino adults with diagnosed HIV, treating the PR-born Hispanics/Latinos as a separate group. Comparing characteristics, including HIV care outcomes, between mainland-US-born, PR-born, and non-US-born Hispanics/Latinos at the national level, can inform tailored HIV prevention and treatment efforts to end the HIV epidemic in Hispanic/Latino communities.

## 2. Materials and Methods

The Medical Monitoring Project (MMP) is an annual cross-sectional survey designed to produce nationally representative estimates of behavioral and clinical characteristics of adults with diagnosed HIV in the United States. MMP data collection is a part of routine public health surveillance, and thus, determined to be nonresearch [9]. Participating areas obtained local institutional review board approval to collect data when required. All participants provided informed consent.

### 2.1. Sampling

MMP uses a two-stage sampling method in which, during the first stage, 23 project areas were sampled from all US states, the District of Columbia, and Puerto Rico [10]. During the second stage, simple random samples of persons with diagnosed HIV aged 18 years and older were drawn for each jurisdiction from the National HIV Surveillance System (NHSS), a census of persons with diagnosed HIV in the United States. 

All sampled states and Puerto Rico participated in MMP. Participating areas included California, Delaware, Florida, Georgia, Illinois, Indiana, Michigan, Mississippi, New Jersey, New York, North Carolina, Oregon, Pennsylvania, Puerto Rico, Texas, Virginia, and Washington. Separately funded cities or jurisdictions include Los Angeles County, San Francisco, Chicago, New York City, Philadelphia, and Houston. For this analysis, we combined three cycle years of MMP data: 2015, 2016, and 2017. Data collection began on June 1 of the cycle year and ended on May 31 of the following calendar year. For each cycle, the sampling frame was adults with diagnosed HIV reported to NHSS as of December 31 of the prior year. The response rate for adults with diagnosed HIV was 40%, 44%, and 46% for the 2015, 2016, and 2017 MMP cycles, respectively. Data were weighted on the basis of known probabilities of selection at state or territory and person levels [11]. Further, data were weighted to adjust for person nonresponse and post-stratified to NHSS population totals by sex, race/ethnicity, and age [12].

### 2.2. Data Collection and Classification

Data were collected via telephone or face-to-face interviews and medical record abstractions from June 2015 to May 2018. Interviews were conducted in English or Spanish. All measures were assessed over the 12 months before the date of the interview unless otherwise noted. Medical records were abstracted at the health care facility the participant identified as their most frequent source of HIV care. Abstracted information included documented prescription of ART, laboratory results, and health care use. Persons born in the mainland US (50 States and Washington DC) were classified as *mainland*-*US-born*, those born in Puerto Rico were classified as *PR-born,* and those born elsewhere were classified as *non-US-born*. Hispanics/Latinos born in US territories other than Puerto Rico were excluded from this analysis due to small cell size. We also categorized PR-born Hispanics/Latinos as currently residing in Puerto Rico or the US mainland. 

### 2.3. Sociodemographic and Behavioral Characteristics

For non-US-born Hispanics/Latinos, age at the time of immigration to the United States was categorized as younger than 18 years old and 18 years or older. The date of diagnosis relative to immigration was calculated by comparing the reported date the person immigrated to the United States with their first HIV diagnosis date. Transgender Hispanics/Latinos were not included in the gender category because of small cell sizes. Binge drinking was defined as drinking five or more alcoholic beverages in a single sitting for men and four or more alcoholic beverages in a single sitting for women in the 30 days prior to the interview. Depression in the past two weeks was assessed using the eight-item Patient Health Questionnaire Depression Scale (PHQ-8) [13], and anxiety in the past two weeks was assessed using the Generalized Anxiety Disorder Scale (GAD-7) [14]. Scales for depression and anxiety were categorized based on clinically meaningful cut points, and detailed algorithms are reported elsewhere [10].

### 2.4. HIV Care and Clinical Outcome Measures

Documentation in the medical record of ART prescription in the 12 months before interview was assessed. Retention in care was defined as documentation of two elements of outpatient HIV care at least 90 days apart in the past 12 months. Outpatient HIV care was defined as documentation in the medical record of any of the following: an encounter with an HIV care provider (could also be self-reported), viral load test result, CD4 test result, HIV resistance test or tropism assay, ART prescription, *Pneumocystis jiroveci* pneumonia prophylaxis, or *Mycobacterium avium* complex prophylaxis. Viral suppression was defined as an HIV RNA level that was undetectable or < 200 copies/mL. Recent viral suppression was defined as virally suppressed at the most recent test, and sustained viral suppression was defined as virally suppressed at all tests in the past 12 months. Adherence to ART doses was assessed using a 3-item scale [15]. Participants were asked how many days they missed at least one dose of their HIV medicines, how often they took their HIV medicines in the way they were supposed to, and how good a job they did at taking their HIV medicines in the way they were supposed to during the 30 days before the interview. Among persons who are taking ART, those who reported no missed doses, always took HIV medicines, and felt they did an excellent job at taking HIV medicines in the way they were supposed to were considered 100% adherent to ART doses.

### 2.5. Analysis

The analytic dataset included 2564 adults with diagnosed HIV that self-identified as Hispanic/Latino, including 944 born in the United States mainland, 634 born in Puerto Rico (of whom 435 currently reside in Puerto Rico and 199 reside in the mainland United States), and 986 born outside the United States. We calculated weighted prevalences with 95% confidence intervals (CI) for demographic, behavioral, and clinical characteristics. Estimates with coefficients of variation ≥0.3 were considered unreliable and were not reported. 

For the analysis, we first reported characteristics of Hispanic/Latino adults with diagnosed HIV by place of birth. Next, we examined differences in selected characteristics and HIV care outcomes by place of birth. We used logistic regression with predicted marginal means to estimate unadjusted prevalence ratios and corresponding 95% CIs using the mainland-US-born as the reference group. Finally, we assessed differences in HIV clinical outcomes by place of residence among PR-born persons using Rao–Scott chi-square tests. For all analyses, associations with a *p*-value of < 0.05 were considered statistically significant.

## 3. Results

### 3.1. Sociodemographic, Behavioral and Clinical Characteristics

Among Hispanic/Latino adults with diagnosed HIV, an estimated 40% were born in the mainland United States, 22% were born in Puerto Rico, and 38% were born outside of the United States (Table 1). Most Hispanics/Latinos with diagnosed HIV were men, regardless of place of birth. Among the PR-born, 67% currently reside in Puerto Rico. Among non-US-born Hispanics/Latinos, nearly half (49%) were born in Mexico. Over three-quarters of non-US-born Hispanics/Latinos immigrated to the United States at age 18 years or older, and most had lived in the United States for 10 or more years. Among non-US-born Hispanics/Latinos, 10% received an HIV diagnosis at least one year before relocating to the United States, 5% received an HIV diagnosis the same year as their arrival, and more than half received an HIV diagnosis 10 or more years after immigrating to the United States. 

A lower proportion of PR-born compared with mainland-US-born and non-US-born Hispanics/Latinos were men (Table 2). PR-born and non-US-born Hispanics/Latinos were older and were less likely to complete a high school diploma or equivalent education than mainland-US-born Hispanics/Latinos. PR-born or non-US-born Hispanics/Latinos were more likely to live in a household at or below the federal poverty level and receive care at a Ryan White HIV/AIDS Program (RWHAP)-funded facility compared with mainland-US-born Hispanics/Latinos. There was a significantly higher proportion of non-US-born Hispanics/Latinos who had any RWHAP coverage in the past 12 months compared with mainland-US-born.

Non-US-born Hispanics/Latinos were less likely to binge drink, use non-injection drugs, and report symptoms of depression and anxiety compared with mainland-US-born Hispanics/Latinos (Table 2). PR-born Hispanics/Latinos were less likely to use non-injection and injection drugs compared with mainland-US-born Hispanics/Latinos. PR-born and non-US-born Hispanics/Latinos were more likely to have an AIDS diagnosis compared with mainland-US-born Hispanics/Latinos.

### 3.2. HIV Outcomes

PR-born Hispanics/Latinos were more likely to have been prescribed ART (94%), retained in care (94%), and virally suppressed at their most recent viral load test (85%) compared with mainland-US-born (79%, 77%, and 65%, respectively) Hispanics/Latinos (Table 3). Non-US-born Hispanics/Latinos were more likely to have been prescribed ART (91%), retained in care (87%), and virally suppressed at their most recent viral load test (80%) compared with mainland-US-born (79%, 77%, and 65%, respectively) Hispanics/Latinos. A higher proportion of PR-born (75%) and non-US-born (74%) Hispanics/Latinos compared with 57% of mainland-US-born Hispanics/Latinos had sustained viral suppression. 

PR-born Hispanics/Latinos who resided in Puerto Rico were more likely to have been prescribed ART (96%), been retained in care (97%), and had sustained viral suppression (78%) compared with those who resided in the US mainland at the time of sampling (91%, 88%, and 68% respectively) (Table 4).

## 4. Discussion

Our findings demonstrate disparities in HIV care outcomes among Hispanics/Latinos with diagnosed HIV by place of birth. Although Hispanics/Latinos born outside of the mainland US had a higher prevalence of AIDS, they were more likely to be on ART, be engaged in care, and virally suppressed compared with mainland-US-born Hispanics/Latinos. Similarly, previous studies reported a higher proportion of non-US-born blacks compared with US-born blacks were retained in HIV medical care and virally suppressed [16]. Sheena and colleagues also reported a higher prevalence of linkage to HIV care after diagnosis among PR-born Hispanics/Latinos residing in Florida, compared with their mainland-US-born counterparts [17]. 

One possible explanation for these outcome disparities may be that more advanced disease among PR-born and non-US-born groups may have resulted in closer medical follow-up that may have led to better HIV care outcomes, such as retention in care. Similarly, a study of patients at two academic medical centers in Massachusetts found that non-US-born persons with HIV more often presented with late-stage disease but were more likely to be linked to care compared with US-born counterparts [18]. 

Another possible reason for poorer HIV outcomes among Hispanics/Latinos born in the US mainland may be differences in the utilization of RWHAP and receipt of care at RWHAP-funded facilities. A lower percentage of mainland-US-born than PR-born and non-US born Hispanics/Latinos received care at RWHAP- funded facilities, which may have contributed to the disparity in HIV care outcomes observed by place of birth. The RWHAP provides funds to HIV clinics to increase access to high-quality HIV care and treatment, including financial assistance for HIV medications and access to supportive services for low-income, uninsured, and underinsured individuals and families affected by HIV [19]. The RWHAP functions as a safety net provider of HIV care and treatment for people who are unable to obtain other forms of health care coverage. Receipt of care at RWHAP-funded facilities has consistently been associated with higher levels of viral suppression among low-income persons [5,20,21]. 

Differences in mental health and substance use are another possible explanation for HIV outcome disparities found between the examined groups. Mainland-US-born Hispanics/Latinos were more likely than PR-born and non-US-born Hispanics/Latinos to report the use of drugs and were more likely than non-US-born Hispanics/Latinos to report symptoms of anxiety and depression. Psychiatric illnesses combined with substance use are associated with poor HIV outcomes [22,23]. Persons with diagnosed HIV who are using drugs are less likely to adhere to treatment and achieve viral suppression [22], and those who are experiencing current symptoms of depression combined with the use of cocaine or methamphetamine were found to have lower odds of virologic suppression [23]. 

We further stratified PR-born Hispanics/Latinos with diagnosed HIV by their current residence and found that PR-born Hispanic/Latinos who resided in Puerto Rico were more likely to be prescribed ART, retained in care, and sustainably virally suppressed compared with those who resided in the mainland US. PR-born Hispanics/Latinos who live on the island were the only groups that reached the national HIV prevention goal of 90% retention in care among persons with HIV [17]. Availability and accessibility of HIV services on the island may be one factor that contributed to favorable outcomes among PR-born Hispanics/Latinos residing on the island. One study found that Puerto Rican adults had better access to health care, after controlling for sociodemographic factors, including income, compared with their counterparts on the US mainland [24]. The difference in HIV care outcomes between PR-born Hispanics/Latinos who reside on the island and in the US mainland needs further research because the migration of Puerto Ricans to the mainland US has accelerated in recent years [25]. Puerto Ricans who relocate to the mainland US may experience interruptions accessing and staying in HIV care. 

Our analysis is subject to limitations. First, MMP’s design is cross-sectional, and thus, causal relationships between sociodemographic, behavioral, and clinical characteristics and HIV outcomes cannot be inferred. Second, MMP’s person-level response rate was low; however, the data were adjusted for nonresponse, which should reduce bias. Third, medical records were abstracted from the facility where the person received the most HIV care, not from all facilities that provided care. Thus, our findings may not be inclusive of all possible medical services the person received.

## 5. Conclusions

Disparities in HIV care outcomes exist among Hispanics/Latinos with diagnosed HIV by place of birth. Factors contributing to favorable HIV care outcomes may include the utilization of RWHAP-funded facilities among PR-born persons and better mental health and less drug use among non-US-born persons. Expanding access and reducing barriers to the use of comprehensive HIV/AIDS programs that provide care to the whole person, including treating mental health and substance use issues, may reduce HIV care outcome disparities and support ending the HIV epidemic in Hispanic/Latino communities.

## Figures and Tables

**Table 1:** Hispanic/Latino adults with diagnosed HIV by place of birth, United States—Medical Monitoring Project, 2015–2018 (n = 2564).

Characteristics	Mainland-US-Born		Puerto Rico-Born		Non-US-Born	
	n	Weighted Column %	n	Weighted Column %	n	Weighted Column %
		(95% CI)		(95% CI)		(95% CI)
**Total**	944	39.8 (33.2–46.4)	634	21.9 (8.8–35.0)	986	38.3 (31.0–45.5)
**Currently reside in Puerto Rico**	-	-	435	66.6 (40.6–92.7)	-	-
**Gender**						
Male	721	80.9 (78.0–83.7)	411	70.6 (66.6–74.6)	776	81.8 (79.0–84.7)
Female	205	19.1 (16.3–22.0)	212	29.4 (25.4–33.4)	185	18.2 (15.3–21.0)
**Age (Years)**						
18–34	254	27.0 (23.5–30.5)	51	7.2 (5.1–9.2)	131	13.1 (10.4–15.7)
35–44	181	21.9 (18.4–25.5)	96	16.0 (12.9–19.1)	265	28.2 (24.7–31.7)
45–54	283	28.7 (25.2–32.3)	229	37.5 (34.4–40.6)	366	36.5 (33.2–39.9)
≥55	226	22.4 (19.2–25.5)	258	39.3 (35.1–43.4)	224	22.2 (19.1–25.3)
**Education**						
<High school	181	16.8 (14.1–19.5)	184	29.2 (25.0–33.3)	341	35.2 (31.9–38.6)
High school diploma or equivalent	244	25.4 (21.9–28.8)	164	26.8 (22.3–31.3)	225	22.8 (19.9–25.7)
>High school	518	57.8 (53.7–61.9)	285	44.0 (39.5–48.5)	415	42.0 (38.1–45.9)
**At or below federal poverty level**	373	39.7 (35.8–43.6)	412	66.2 (62.0–70.4)	448	47.9 (44.1–51.8)
**Speaks language other than English at home**	532	56.3 (52.0–60.6)	607	96.6 (93.8–99.4)	875	88.9 (86.2–91.6)
**Speaks English well or very well**	924	98.1 (97.0–99.2)	334	53.2 (40.8–65.6)	628	64.2 (60.2–67.9)
**Received care at a Ryan White HIV/AIDS Program-funded facility ^a,b^**	631	68.9 (64.5–73.4)	584	91.0 (83.6–98.5)	742	77.8 (73.4–82.3)
**Had any Ryan White coverage ^c^**	402	40.4 (36.2–44.7)	257	43.7 (37.1–50.3)	603	62.9 (58.9–66.9)
**Country of birth**						
Caribbean	-	-	-	-	153	15.4 (12.1–18.7)
Central America	-	-	-	-	177	17.1 (14.2–20.0)
Mexico	-	-	-	-	474	48.6 (43.6–53.6)
South America	-	-	-	-	157	16.2 (13.2–19.2)
Others	-	-	-	-	24	2.7 (1.3–4.0)
**Age at migration (Years)**						
<18	-	-	-	-	231	24.2 (21.1–27.4)
≥18	-	-	-	-	750	75.8 (72.6–78.9)
**Time living in the United States (Years)**						
<5	-	-	-	-	44	5.1 (3.2–6.9)
5–9	-	-	-	-	51	4.2 (3.0–5.5)
≥10	-	-	-	-	886	90.7 (88.6–92.8)
**Date of diagnosis relative to immigration**						
≥5 years before arrival	-	-	-	-	51	5.1 (3.5–6.6)
1–4 years before arrival	-	-	-	-	44	4.7 (3.1–6.3)
Same year as arrival	-	-	-	-	42	5.0 (3.4–6.7)
1–4 years after arrival	-	-	-	-	136	13.7 (11.3–16.1)
5–9 years after arrival	-	-	-	-	191	19.0 (16.1–21.8)
≥10 years after arrival	-	-	-	-	516	52.5 (49.0–56.1)

Notes: CI, Confidence Interval; all percentages are weighted; time period is 12 months prior to interview unless otherwise noted; all measures are self-reported unless otherwise noted. ^a^ Any Parts A, B, C, D, or F funding. ^b^ Documented in medical record. ^c^ Includes AIDS Drug Assistance Program (ADAP) and other RWHAP support.

**Table 2:** Comparison of selected demographic, behavioral, and clinical characteristics of Hispanic/Latino adults with diagnosed HIV by place of birth, United States— Medical Monitoring Project, 2015–2018 (n = 2564).

	Weighted Row % (95% CI)	Prevalence Ratio (95% CI)	*p*-Value ^a^
**Gender (Male)**			
Mainland-US-born	80.9 (78.0–83.7)	Ref.	--
Puerto Rico-born	70.6 (66.6–74.6)	0.87 (0.82–0.93)	<0.0001
Non-US-born	81.8 (79.0–84.7)	1.01 (0.96–1.06)	0.641
**Age (18–44 Years)**			
Mainland-US-born	48.9 (44.9–53.0)	Ref.	-
Puerto Rico-born	23.2 (19.8–26.6)	0.47 (0.40–0.56)	<0.0001
Non-US-born	41.3 (37.5–45.0)	0.84 (0.75–0.95)	0.0064
**Education (<High school)**			
Mainland-US-born	16.8 (14.1–19.5)	Ref.	-
Puerto Rico-born	29.2 (25.0–33.3)	1.74 (1.41–2.14)	<0.0001
Non-US-born	35.2 (31.9–38.6)	2.10 (1.74–2.53)	<0.0001
**At or below federal poverty level**			
Mainland-US-born	39.7 (35.8–43.6)	Ref.	-
Puerto Rico-born	66.2 (62.0–70.4)	1.67 (1.49–1.87)	<0.0001
Non-US-born	47.9 (44.1–51.8)	1.21 (1.07–1.36)	0.0022
**Speaks language other than English at home**			
Mainland-US-born	56.3 (52.0–60.6)	Ref.	-
Puerto Rico-born	96.6 (93.8–99.4)	1.72 (1.60–1.84)	<0.0001
Non-US-born	88.9 (86.2–91.6)	1.58 (1.46–1.71)	<0.0001
**Speaks English well or very well**			
Mainland-US-born	98.1 (97.0–99.2)	Ref.	-
Puerto Rico-born	53.2 (40.8–65.6)	0.54 (0.43–0.68)	<0.0001
Non-US-born	64.2 (60.5–67.9)	0.65 (0.62–0.69)	<0.0001
**Received care at a Ryan White HIV/AIDS Program-funded facility ^b,c^**			
Mainland-US-born	68.9 (64.5–73.4)	Ref.	--
Puerto Rico-born	91.0 (83.6–98.5)	1.30 (1.19–1.41)	<0.0001
Non-US-born	77.8 (73.4–82.3)	1.11 (1.05–1.19)	0.0008
**Had any Ryan White coverage ^d^**			
Mainland-US-born	40.4 (36.2–44.7)	Ref.	-
Puerto Rico-born	43.7 (37.1–50.3)	1.06 (0.89–1.27)	0.5296
Non-US-born	62.9 (58.9–66.9)	1.57 (1.41–1.74)	<0.0001
**Binge drinking, past 30 days ^e^**			
Mainland-US-born	21.9 (18.5–25.2)	Ref.	--
Puerto Rico-born	18.9 (8.7–29.2)	0.87 (0.49–1.52)	0.5886
Non-US-born	15.7 (13.2–18.3)	0.72 (0.58–0.90)	0.0041
**Non-injection drug use**			
Mainland-US-born	37.4 (33.3–41.4)	Ref.	-
Puerto Rico-born	17.7 (14.7–20.7)	0.47 (0.39–0.58)	<0.0001
Non-US-born	14.2 (11.6–16.7)	0.38 (0.31–0.47)	<0.0001
**Injection drug use**			
Mainland-US-born	4.3 (2.5–6.1)	Ref.	-
Puerto Rico-born	1.5 (0.7–2.3)	0.35 (0.18–0.70)	0.0066
Non-US-born ^f^	-	-	-
**Major or other depression, past 2 weeks ^g^**			
Mainland-US-born	27.6 (24.0–31.2)	Ref.	-
Puerto Rico-born	22.2 (18.5–25.9)	0.80 (0.66–0.98)	0.0521
Non-US-born	16.9 (14.1–19.8)	0.61 (0.49–0.76)	<0.0001
**Moderate to severe anxiety, past 2 weeks ^h^**			
Mainland-US-born	20.7 (17.5–24.0)	Ref.	-
Puerto Rico-born	19.1 (16.6–21.6)	0.92 (0.75–1.13)	0.4509
Non-US-born	11.2 (8.9–13.5)	0.54 (0.42–0.70)	<0.0001
**Stage 3 classification (AIDS)**			
Mainland-US-born	50.6 (46.5–54.7)	Ref.	--
Puerto Rico-born	56.5 (52.2–60.8)	1.12 (1.00–1.24)	0.0352
Non-US-born	58.5 (54.8–62.1)	1.16 (1.04–1.28)	0.0056

Notes: CI, Confidence Interval; all percentages are weighted; time period is 12 months prior to interview unless otherwise noted; all measures are self-reported unless otherwise noted. ^a^ *p*-value compared with US-born. ^b^ Documented in medical record. ^c^ Any Parts A, B, C, D, or F funding. ^d^ Includes AIDS Drug Assistance Program (ADAP) and other RWHAP support. ^e^ Drinking ≥5 alcoholic beverages in a single sitting for men, and ≥4 for women. ^f^ Coefficient of variation >0.3. Estimate is unstable. ^g^ Using the Patient Health Questionnaire Depression Scale (PHQ-8). ^h^ Using the Generalized Anxiety Disorder Scale (GAD-7).

**Table 3:** HIV outcomes among Hispanic/Latino adults with diagnosed HIV by place of birth, United States—Medical Monitoring Project, 2015–2018 (n = 2564).

	Weighted Row % (95% CI)	Prevalence Ratio (95% CI)	*p*-Value ^a^
**ART prescription**			
Mainland-US-born	78.6 (74.5–82.6)	Ref.	-
Puerto Rico-born	94.0 (91.8–96.2)	1.20 (1.13–1.26)	<0.0001
Non-US-born	90.9 (88.4–93.4)	1.16 (1.09–1.23)	<0.0001
**100% adherent to ART doses, past 30 days ^b,c^**			
Mainland-US-born	38.6 (34.5–42.7)	Ref.	-
Puerto Rico-born	41.5 (36.2–46.9)	1.08 (0.91–1.27)	0.37
Non-US-born	44.7 (41.0–48.3)	1.16 (1.01–1.33)	0.03
**Retention in care ^d^**			
Mainland-US-born	77.3 (73.1–81.4)	Ref.	-
Puerto Rico-born	94.3 (90.8–97.8)	1.22 (1.15–1.30)	<0.0001
Non-US-born	87.3 (84.5–90.1)	1.13 (1.06–1.20)	<0.0001
**Recent viral suppression ^e^**			
Mainland-US-born	65.1 (60.8–69.4)	Ref.	-
Puerto Rico-born	84.6 (80.8–88.5)	1.30 (1.20–1.40)	<0.0001
Non-US-born	80.1 (76.8–83.4)	1.23 (1.14–1.32)	<0.0001
**Sustained viral suppression ^f^**			
Mainland-US-born	57.3 (53.1–61.5)	Ref.	-
Puerto Rico-born	74.6 (70.5–78.8)	1.30 (1.19–1.42)	<0.0001
Non-US-born	73.8 (70.4–77.2)	1.29 (1.18–1.40)	<0.0001

Notes: CI, Confidence Interval; all percentages are weighted; time period is 12 months prior to interview unless otherwise noted; all measures documented in medical record unless otherwise noted. ^a^ *p*-value compared with US-born. ^b^ Self-reported. ^c^ Among persons taking ART. ^d^ 2 visits at least 90 days apart in the past 12 months. ^e^ Most recent viral load that was undetectable or <200 copies/mL. ^f^ All viral loads in the past 12 months undetectable or <200 copies/mL.

**Table 4:** HIV outcomes among Puerto Rico-born Hispanic/Latino adults with diagnosed HIV by current residency, United States—Medical Monitoring Project, 2015–2018 (n = 634).

Characteristics	Reside in PR (n = 435)		Reside in Mainland US (n = 199)		*p*-Value
	n	Weighted Row % (95% CI)	n	Weighted Row % (95% CI)	
**ART prescription**	419	95.5 (94.8–96.2)	184	91.0 (85.5–96.4)	0.0066
**100% adherent to ART doses, past 30 days ^a^**	170	42.4 (35.8–49.0)	78	39.7 (31.9–47.5)	0.5902
**Retention in care ^b^**	424	97.2 (95.2–99.1)	178	88.4 (82.1–94.7)	0.0192
**Recent viral suppression ^c^**	381	87.9 (85.1–90.6)	158	78.1 (71.0–85.2)	<0.0001
**Sustained viral suppression ^d^**	335	78.2 (75.4–80.9)	136	67.6 (60.1–75.0)	<0.0001

Notes: CI, Confidence Interval; all percentages are weighted; time period is 12 months prior to interview unless otherwise noted; all measures documented in medical record unless otherwise noted. ^a^ Among persons taking ART. ^b^ 2 visits at least 90 days apart in the past 12 months. ^c^ Most recent viral load that was undetectable or <200 copies/mL. ^d^ All viral loads in the past 12 months undetectable or <200 copies/mL.

## References

[1] Centers for Disease Control and Prevention (CDC) Estimated HIV Incidence and Prevalence in the United States, 2010–2016. HIV Surveillance Supplemental Report, 2019, 24 (No. 1). http://www.cdc.gov/hiv/library/reports/hiv-surveillance.html.

[2] Centers for Disease Control and Prevention (CDC) Monitoring Selected National HIV Prevention and Care Objectives by Using HIV Surveillance Data United States and 6 Dependent Areas, 2017. HIV Surveillance Supplemental Report, 2019, 24 (No. 3). http://www.cdc.gov/hiv/library/reports/hiv-surveillance.html.

[3] Chen N.E., Gallant J.E., Page K.R. (2012). A systematic review of HIV/AIDS survival and delayed diagnosis among Hispanics in the United States. J. Immigr. Minor. Health.

[4] Sheehan D.M., Trepka M.J., Dillon F.R. (2015). Latinos in the United States on the HIV/AIDS care continuum by birth country/region: A systematic review of the literature. Int. J. STD AIDS.

[5] The White House (2015). National HIV/AIDS Strategy for the United States: Updated to 2020.

[6] Espinoza L., Hall H.I., Selik R.M., Hu X. (2008). Characteristics of HIV infection among Hispanics, United States 2003–2006. J. Acquir. Immune Defic. Syndr..

[7] Prosser A.T., Tang T., Hall H.I. (2012). HIV in persons born outside the United States, 2007-2010. JAMA.

[8] Demeke H.B., Luo Q., Beer L., Weiser J. US-born and non-US-born adults with diagnosed HIV in the United States—2015, Medical Monitoring Project (MMP). Proceedings of the National HIV Prevention Conference.

[9] Centers for Disease Control and Prevention (CDC) (2010). Distinguishing Public Health Research and Public Health Nonresearch. https://www.cdc.gov/od/science/integrity/docs/cdc-policy-distinguishing-public-health-research-nonresearch.pdf.

[10] Centers for Disease Control and Prevention (CDC) (2019). Behavioral and Clinical Characteristics of Persons with Diagnosed HIV Infection—Medical Monitoring Project, United States, 2016 Cycle (June 2016–May 2017). HIV Surveillance Special Report. Revised edition. https://www.cdc.gov/hiv/library/reports/hiv-surveillance.html.

[11] The American Association for Public Opinion Research (2011). Standard Definitions: Final Dispositions of Case Codes and Outcome Rates for Surveys. https://www.aapor.org/Standards-Ethics/Standard-Definitions-(1).aspx.

[12] Heeringa S.G., West B.T., Berglund P.A. (2010). Applied Survey Data Analysis.

[13] Kroenke K., Strine T.W., Spitzer R.L., Williams J.B., Berry J.T., Mokdad A.H. (2009). The PHQ-8 as a measure of current depression in the general population. J. Affect. Disord..

[14] Spitzer R.L., Kroenke K., Williams J.B., Lowe B. (2006). A brief measure for assessing Generalized Anxiety Disorder: The GAD-7. Arch. Intern. Med..

[15] Wilson I.B., Lee Y., Michuad J., Fowler F.J., Rogers W.H. (2016). Validation of a New Three-Item Self-Report Measure for Medication Adherence. AIDS Behav..

[16] Demeke H.B., Johnson A.S., Zhu H., Gant Z., Duffus W.A., Dean H.D. (2018). HIV Infection-related care outcomes among US-born and non–US-born blacks with diagnosed HIV in 40 US Areas, National HIV Surveillance System, 2016. Int. J. Environ. Res. Public Health.

[17] Sheehan D.M., Cosner C., Fennie K.P., Gebrezgi M.T., Cyrus E., Maddox L.M., Levison J.H., Spencer E.C., Niyonsenga T., Trepka M.J. (2018). Role of country of birth, testing site, and neighborhood characteristics on nonlinkage to HIV care among Latinos. AIDS Patient Care STDS.

[18] Levison J.H., Regan S., Khan I., Freedberg K.A. (2017). Foreign-born status as a predictor of engagement in HIV care in a large US metropolitan health system. AIDS Care.

[19] U.S. Department of Health and Human Services (2018). Overview: Ryan White HIV/AIDS Program Clients, 2016. https://hab.hrsa.gov/about-ryan-white-hivaids-program/about-ryan-white-hivaids-program.

[20] Weiser J., Beer L., Frazier E.L., Patel R., Dempsey A., Hauck H., Skarbinski J. (2015). Service delivery and patient outcomes in Ryan White HIV/AIDS Program-funded and -nonfunded health care facilities in the United States. JAMA Intern. Med..

[21] Bradley H., Viall A.H., Wortley P.M., Dempsey A., Hauck H., Skarbinski J. (2016). Ryan White HIV/AIDS program assistance and HIV treatment outcomes. Clin. Infect. Dis..

[22] Aralis H.J., Shoptaw S., Brookmeyer R., Ragsdale A., Bolan R., Gorbach P.M. (2018). Psychiatric illness, substance use, and viral suppression among HIV-positive men of color who have sex with men in Los Angeles. AIDS Behav..

[23] Fojo A.T., Lesko C.R., Calkins K.L., Moore R.D., McCaul M.E., Hutton H.E., Mathews W.C., Crane H., Christopoulos K., Cropsey K. (2019). Do symptoms of depression interact with substance use to affect HIV continuum of care outcomes?. AIDS Behav..

[24] Portela M., Sommers B.D. (2015). On the Outskirts of National Health Reform: A Comparative Assessment of Health Insurance and Access to Care in Puerto Rico and the United States. Milbank Q..

[25] Krogstad J.M. (2015). Puerto Ricans Leave in Record Numbers for Mainland U.S..

